# Depression and Catechol-O-methyltransferase (*COMT*) genetic variants are associated with pain in Parkinson’s disease

**DOI:** 10.1038/s41598-017-06782-z

**Published:** 2017-07-24

**Authors:** Chin-Hsien Lin, K. Ray Chaudhuri, Jun-Yu Fan, Chia-I. Ko, Alexandra Rizos, Chia-Wen Chang, Han-I. Lin, Yih-Ru Wu

**Affiliations:** 1 Department of Neurology, National Taiwan University Hospital, College of Medicine, National Taiwan University, Taipei, Taiwan; 2 Maurice Wohl Clinical Neuroscience Institute and NIHR Biomedical Research Centre, Institute of Psychiatry, Psychology and Neuroscience, King’s College Hospital, London, UK; 3 Department of Nursing, Chang Gung University of Science and Technology, Taoyuan, Taiwan; 4 Nursing Department, Linkou Chang Gung Memorial Hospital, Taoyuan, Taiwan; 5 National Parkinson Foundation Centre of Excellence, King’s College Hospital, London, London UK; 6 Department of Neurology, Chang Gung Memorial Hospital, Chang Gung University College of Medicine, Linkou, Taiwan

## Abstract

Pain is a distressing symptom of Parkinson disease (PD). We aim to determine whether the genetic variants of chronic pain-related genes contribute to pain in PD patients. We included 418 PD patients and evaluated pain severity on King’s PD pain scale. We genotyped rs6267, rs6269, rs4633, rs4818 and rs4680 of *COMT*, rs6746030 of *SCN9A*, and rs1799971 of *OPRM1*. In total, 193 participants (46.2%) experienced pain. Compared to pain-free PD patients, PD patients with pain had an earlier age of onset, longer disease duration, and higher depression and motor severity (*P* < 0.01). The frequencies of *COMT* rs4680 “A” allele were higher in PD patients with pain than those without pain (46.1% vs. 31.1%, *P* < 0.01). Pain severity was significantly associated with disease duration (*P* = 0.02), and *COMT* rs6267 T allele (*P* < 0.01). We stratified PD by status of depression and the association between *COMT* rs6267 “GT” genotype and pain severity remained significant (*P* < 0.01). Furthermore, pain severity was significantly higher in participants having *COMT* rs4680 “GG” and “GA” genpotypes than those having “AA” genotype (*P* = 0.04). We concluded that depression and *COMT* rs4680 “GG” and “GA” genotypes and *COMT* rs6267 “GT” genotype contribute to pain in PD patients.

## Introduction

Parkinson disease (PD) is a multidimensional neurodegenerative disorder with both motor and nonmotor symptoms^1^. Sensory complaints, particularly pain, are one of the most common nonmotor symptoms; nearly 43% of patients with PD experience primary pain related to dysfunction of the nociceptive system in early PD stages, in which motor symptoms are not yet prominent^2, 3^. Furthermore, patients with PD present a reduction of the pain threshold for both thermal and pain stimulation^4, 5^. The pathophysiological mechanisms of PD pain arise from dysfunction of the peripheral and central compartments of the nociceptive pathways^6–8^. Treatment with levodopa could not only relieve pain but also increase the pain threshold in patients with PD^9^; this observation confirms that the mesolimbic dopamine pathway and nucleus accumbens have profound roles in pain modulation^10^. Because substantial interindividual variability can be observed in pain perception and response to medications, the single nucleotide polymorphisms (SNPs) of several genes involved in the nociceptive pathways have recently been implicated in pain sensitivity in patients with chronic pain syndromes^11–13^. Notably, among these genes, catechol-O-methyltransferase gene (*COMT*) encodes a key metabolizing enzyme that degrades catecholamines including dopamine, epinephrine, and norepinephrine^11^. It therefore contributes to maintaining the homeostasis for a variety of pivotal biological systems including pain perception, mood, and responses to both physical and emotional stressors. A functional SNP in codon 158 (Val158Met) of *COMT* has been shown to modulate pain perception and contribute to differences of pain perception^14^. Haplotypes composed of combinations of alleles of rs6269, rs4633, rs4818, and rs4680 of the *COMT* gene were previously shown to affect COMT activity and grouped COMT function into three haploblocks based on pain response, which covered about 96% of the people tested^15^. Furthermore, COMT is also a key enzyme that metabolizes dopamine, which is a pathognomonic neurotransmitter deficiency in patients with PD. Thus, the examination of the role of *COMT* polymorphisms and haplotypes with pain perception and severity is of considerable importance in patients with PD.

Despite the accumulating evidence regarding the complex neural circuits involved in the pathophysiology of pain in patients with PD, data on the specific genetic and molecular mechanisms potentially associated with PD pain remain limited. Our study was based on the hypothesis that PD-related pain shared part of the common mechanism with other chronic pain syndromes, and we assumed that pain-related genes identified for these syndromes also play roles in the risk of PD pain. Therefore, in this study, we explored the contribution of SNPs of nociception-related genes, including *COMT* and the μ-opioid receptor (*OPRM1*) and sodium channel Nav1.7 (*SCN9A*), to the risk and severity of pain in PD. We genotyped rs6267, rs6269, rs4633, rs4818 and rs4680 of the *COMT* gene, rs6746030 of the *SNCA9A* gene, and rs1799971 of the *OPRM1* gene. Because haplotypes formed by rs6269, rs4633, rs4818, and rs4680 of *COMT* gene constituted central *COMT* locus haploblock that is associated with pain response, *COMT* haplotypes were also analyzed in the multivariate regression model to examine the independent effects of various clinical and genetic risk factors contributing to pain in patients with PD.

## Results

**Figure 1:**
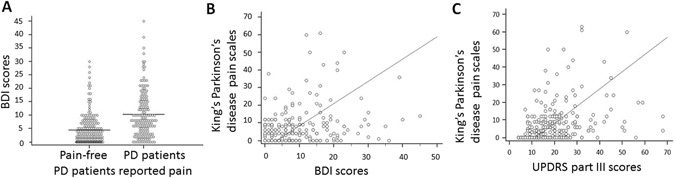
Clinical characteristics correlated with pain susceptibility and severity in patients with PD. (**A**) PD Patients with with pain had higher BDI scores than did those without pain. Pain intensity was defined by KPPS scores and was significantly correlated with depression severity (r = 0.201, *P* = 0.003; (**B**)) and motor disability (r = 0.311, *P* < 0.001; (**C**)) in patients with PD.

We initially recruited 656 patients who received clinical diagnoses of PD; among them, 238 patients were excluded because they did not meet the inclusion criteria or were unwilling to participate in this study. In total, 418 PD patients were enrolled. Of them, 193 (46.2%) reported pain (average age, 64.5 ± 11.1 years; 98 men [50.8%]), whereas the remaining 225 did not (average age, 64.7 ± 13.3 years; 131 men [58.2%]). Compared to pain-free PD patients, those reported pain had earlier age of motor symptom onset (58.5 ± 11.2 vs. 61.4 ± 10.8 years, *P* < 0.01), longer disease duration (8.2 ± 4.3 vs. 5.5 ± 3.8 years, *P* < 0.01), higher BDI scores (10.6 ± 8.9 vs. 4.6 ± 3.8, *P* < 0.01; Fig. 1A), higher Hoehn Yahr stage (2.2 ± 1.0 vs. 1.9 ± 0.9, *P* < 0.01), and higher UPDRS part III scores (21.7 ± 11.6 vs. 18.1 ± 10.6, *P* < 0.01) and total scores (43.8 ± 19.6 vs. 34.2 ± 14.7, *P* < 0.01). The patients’ clinical characteristics and medical comorbidities are listed in Supplementary Table 1. Among the 193 PD patients who reported pain, 105 had musculoskeletal pain (46.7%), 24 had chronic neuropathic pain (10.7%), 31 had motor fluctuation-related pain (13.8%), 32 had nocturnal pain or restless legs syndrome (14.2%), 10 had orofacial pain (4.4%), 9 had limb edema- or swelling-related pain (4.0%), and 14 had radicular pain (6.2%; Supplementary Fig. 1). Only a few patients with pain were periodically administered analgesics or benzodiazepine. Patients who had multiple overlapping pain reports (n = 27, five patients had combined musculoskeletal pain and motor fluctuation related pain, four patients on had combined musculoskeletal pain and symptoms of restless legs syndrome, five patients had combined chronic pain syndrome and motor fluctuation related pain, twelve patients had combined chronic pain, motor fluctuation related pain and restless legs syndrome, one patient had combined musculoskeletal pain and orolingual pain) were collapsed into the major concerning domain in the KPPS.

We first investigated the relationship between the presence of pain and individual clinical risk factors to analyse the effects of onset age, sex, disease duration, depression severity, and anti-PD drug consumption on the pain susceptibility using either the Pearson product–moment correlation coefficient or Spearman rank correlation coefficient. We noted that among the clinical variables, depression severity, defined by Beck depression inventory (BDI) scores, was significantly correlated with the intensity of pain perception, defined by the King’s PD pain scale (KPPS) (r = 0.201, *P* = 0.003; Fig. 1B). In addition, the severity of motor symptoms, defined by the Unified Parkinson’s Disease Rating Scale (UPDRS) part III motor subscores, were significantly correlated with the intensity of pain perception (r = 0.311, *P* < 0.001; Fig. 1C).

**Figure 2:**
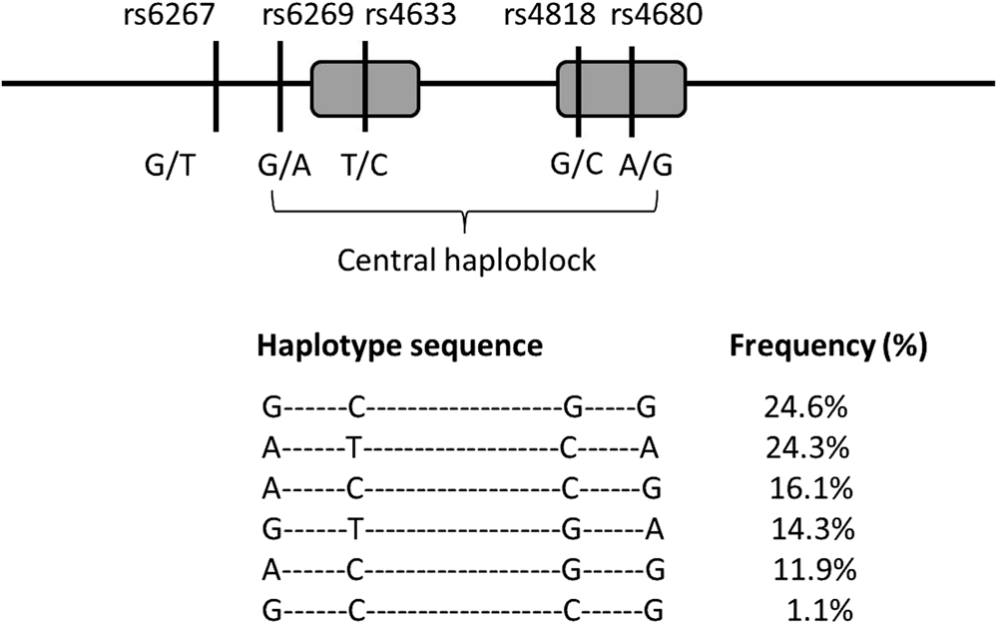
Schematic diagram of *COMT* genomic organization, SNP positions and percentage distribution of *COMT* haplotypes. The sequence of alleles in each haplotype for central haploblock of *COMT* gene reflects the order of occurrence from 5′ to 3′ in the *COMT* gene (SNPs: rs6269, rs4633, rs4818 and rs4680, respectively).

The genotypes and estimated odds ratio of the examined SNPs of *COMT*, *SNCA9A*, and *OPRM1* in relation to the risk of pain in patients with PD are listed in Table 1. The frequency of the *COMT* rs4680 “GA” and “AA” genotypes (Val/Met heterozygotes and Met/Met homozygotes) was higher in PD patients with pain than pain-free PD patients (46.1% vs. 31.1%, *P* < 0.01; it remained significant even after Bonferroni correction), and the presence of the “A” allele increased the susceptibility of pain in patients with PD (odds ratio [OR] = 1.70, 95% confidence interval [CI] = 1.24–2.40, *P* < 0.01). The genotype or allele frequency of *COMT* rs6267, *SNCA9A* rs6746030, and *OPRM1*rs1799971 did not differ between PD patients with pain and pan-free patients. Furthermore, because haplotypes formed by rs6269, rs4633, rs4818, and rs4680 of *COMT* gene constituted central *COMT* locus haploblock that is associated with pain response^15^, we examined whether combinations of alleles (haplotypes) that were formed by the aforementioned four SNPs of *COMT* affect the susceptibility to pain in patients with PD. Three *COMT* haploblocks were previously determined in a prior study by linkage disequilibrium analysis^15^. There are two major forms of COMT enzyme: membrane bound and soluble forms. There were three *COMT* SNPs (rs4633, rs4818 and rs4680) located within the central coding region for both membraneous and soluble forms of COMT. Because the association with pain sensitivity was previously observed for SNPs rs6269 and rs4818, located in the central *COMT* locus haploblock, in patients with a chronic pain syndrome^15^, we therefore focused our analyses on the haplotype covering this central haplobolck, reflecting the order of occurrence from 5′ to 3′ in the *COMT* gene as rs6269, rs4633, rs4818 and rs4680, respectively (Fig. 2). Six haplotypes out of possible 16 were detected from these four SNPs with the most frequent haplotype (Haplotype 1, 24.6%) composed of the most frequent alleles for SNPs rs4633 and rs4680 and the least frequent alleles for SNPs rs6269 and rs4818 (G_C_G_G for SNPs rs6269, rs4633, rs4818 and rs4680, respectively, Fig. 2). The second major haplotype (Haplotype 2, 24.3%) was composed of the most frequent alleles for SNPs rs6269 and rs4818 and the least frequent alleles for SNPs rs4633 and rs4680 (A_T_C_A). The third haplotype (Haplotype 3, 16.1%) was composed of a combination of the most frequent alleles for all SNPs (A_C_C_G). The fourth major haplotype (Haplotype 4, 14.3%) composed of the least frequent alleles for all markers (G_T_G_A). The fifth major haplotype (Haplotype 5, 11.9%) composed of the described alleles of the four markers (A_C_G_G) and the sixth haploptye (Haplotype 6, 1.1%) composed of the described alleles of the four markers (G_C_C_G). These six haplotypes accounted for 92.3% of all detected haplotypes in our studied population.

**Table 1:** Distribution of genotypes and estimated OR of nonsynonymous variants in relation to risk of pain in patients with PD.

	Pain-free PD patients N = 225	PD patients with pain N = 193	OR (95% CI)	*P* value^a^
*COMT rs6267 c*.*214G* > *T*(*p*.*A72S*)				
*GG*	214 (95.1%)	186 (96.4%)		
*GT*	11 (4.9%)	7 (3.6%)		
*TT*	0 (0)	0 (0)		
*T vs*. *G allele*			0.73 (0.29–1.93)	*P* = 0.78
*COMT rs6269 intronic variant G* > *A*				
*GG*	125 (55.6%)	114 (59.1%)		
*GA*	97 (43.1%)	78 (40.4%)		
*AA*	3 (1.3%)	1 (0.5%)		
*A vs*. *G allele*			0.88(0.63–1.22)	*P* = 0.45
*COMT rs4633 c*.*435C* > *T* (*p*.*H62H*)				
*CC*	134 (59.6%)	98 (50.8%)		
*CT*	74 (32.9%)	78 (40.4%)		
*TT*	17 (7.5%)	17 (8.8)		
*T vs*. *C allele*			1.29 (0.95–1.76)	*P* = *0*.*10*
*COMT rs4818 c*.*C657G* (*p*.*L136L*)				
*CC*	102 (45.3%)	92 (47.7%)		
*CG*	98 (43.5%)	75 (38.9%)		
*GG*	25 (11.2%)	26 (13.4%)		
*G vs*. *C allele*			1.19 (0.90–1.59)	*P* = *0*.*21*
*COMT rs4680 c*. *472G* > *A* (*p*.*V158M*)				
*GG*	155 (67.9%)	104 (53.9%)		
*GA*	59 (26.2%)	73 (37.8%)		
*AA*	11 (4.9%)	16 (8.3%)		
*A vs*. *G allele*			1.70 (1.24–2.40)	*P* < 0.01*
*SCN9A rs6746030 c*.*3448G* > *A* (*p*.*R1150W*)				
*GG*	213 (94.7%)	185 (95.9%)		
*GA*	11 (4.9%)	6 (3.1%)		
*AA*	1 (0.4%)	2 (1.0%)		
*A vs*. *G allele*			0.87 (0.36–2.10)	*P* = 0.86
*OPRM1 rs1799971 c*.*A118G* (*p*.*N40D*)				
*AA*	110 (48.9%)	95 (49.2%)		
*AG*	84 (37.3%)	67 (34.7%)		
*GG*	31 (13.8%)	31 (16.1%)		
*G vs*. *A allele*			0.96 (0.72–1.29)	*P* = 0.81

PD, Parkinson disease; OR, odds ratio; CI, confidence interval.

^a^Chi-Square test or Fisher exact test (when frequency <5) were applied.

*P value remained significant after Bonferroni correction with conservative *P* = 0.05/4 = 0.0125.

To explore the independent effects of various clinical and genetic risk factors contributing to pain susceptibility in PD patients, we applied a multivariate stepwise regression model that simultaneously considered the effects of onset age, disease duration, sex, depression, motor symptom severity, exonic nonsynonymous SNPs of candidate genes and haplotypes of the *COMT* gene. The results showed that only depression severity was significantly correlated with pain susceptibility in patients with PD (Beta = 0.018, *P* < 0.01 for BDI scores; adjusted R^2^ = 0.451 and *P* = 0.002; Table 2).

**Table 2:** Multiple stepwise regression analysis of factors correlated with susceptibility of pain in patients with PD (n = 418).

Independent variables	Coefficient	Std. Error	*R* partial	*t*	*P* value
(Constant)	0.568				
Age at onset (years)	−0.004	0.004	−0.085	−1.140	0.26
Disease duration (years)	0.014	0.008	0.129	1.741	0.08
Gender	0.011	0.068	0.012	0.156	0.88
BDI scores	0.018	0.004	0.295	4.134	<0.01**
UPDRS part III scores	0.003	0.004	0.058	0.773	0.44
rs6267 T allele	0.039	0.163	0.018	0.244	0.81
rs4680 A allele	0.030	0.129	0.017	0.233	0.82
rs6746030 A allele	0.055	0.136	0.030	0.407	0.68
rs1799971 G allele	0.017	0.048	0.027	0.362	0.72
Haplotype 1	−0.175	0.106	−0.122	−1.649	0.10
Haplotype 2	0.198	0.509	0.0291	0.389	0.70
Haplotype 3	−0.212	0.117	−0.134	−1.805	0.07
Haplotype 4	−0.118	0.169	−0.0521	−0.698	0.49
Haplotype 5	−0.300	0.486	−0.046	−0.617	0.54
Haplotype 6	0.478	0.472	0.0756	1.014	0.31

In this model, the presence of pain in patients with PD was set as the dependent variable and the onset age of PD motor symptoms, disease duration, sex, BDI scores, UPDRS part III scores in the on state of PD, and minor allele frequency of pain-related candidate genes in this study were set as the independent variables (adjusted R^2^ = 0.451 and *P* = 0.002). R: correlation coefficient based on the model of logistic regression; *t*: *t* value for the coefficient of each parameter in the model; *p*: for *R* or *t*. BDI, Beck Depression Inventory; UPDRS, Unified PD rating scale.

The sequence of alleles in each haplotype for central haploblock of *COMT* gene reflects the order of occurrence from 5′ to 3′ in the *COMT* gene (SNPs: rs6269, rs4633, rs4818 and rs4680, respectively). Six haplotypes out of possible 16 were detected from these four SNPs with the most frequent haplotype (Haplotype 1, 24.6%) composed of the most frequent alleles for SNPs rs4633 and rs4680 and the least frequent alleles for SNPs rs6269 and rs4818 (G_C_G_G for SNPs rs6269, rs4633, rs4818 and rs4680, respectively). The second major haplotype (Haplotype 2, 24.3%) was composed of the most frequent alleles for SNPs rs6269 and rs4818 and the least frequent alleles for SNPs rs4633 and rs4680 (A_T_C_A). The third haplotype (Haplotype 3, 16.1%) was composed of a combination of the most frequent alleles for all SNPs (A_C_C_G). The fourth major haplotype (Haplotype 4, 14.3%) composed of the least frequent alleles for all markers (G_T_G_A). The fifth major haplotype (Haplotype 5, 11.9%) composed of the described alleles of the four markers (A_C_G_G) and the sixth haploptye (Haplotype 6, 1.1%) composed of the described alleles of the four markers (G_C_C_G). These six haplotypes accounted for 92.3% of all detected haplotypes in our studied population.

**Figure 3:**
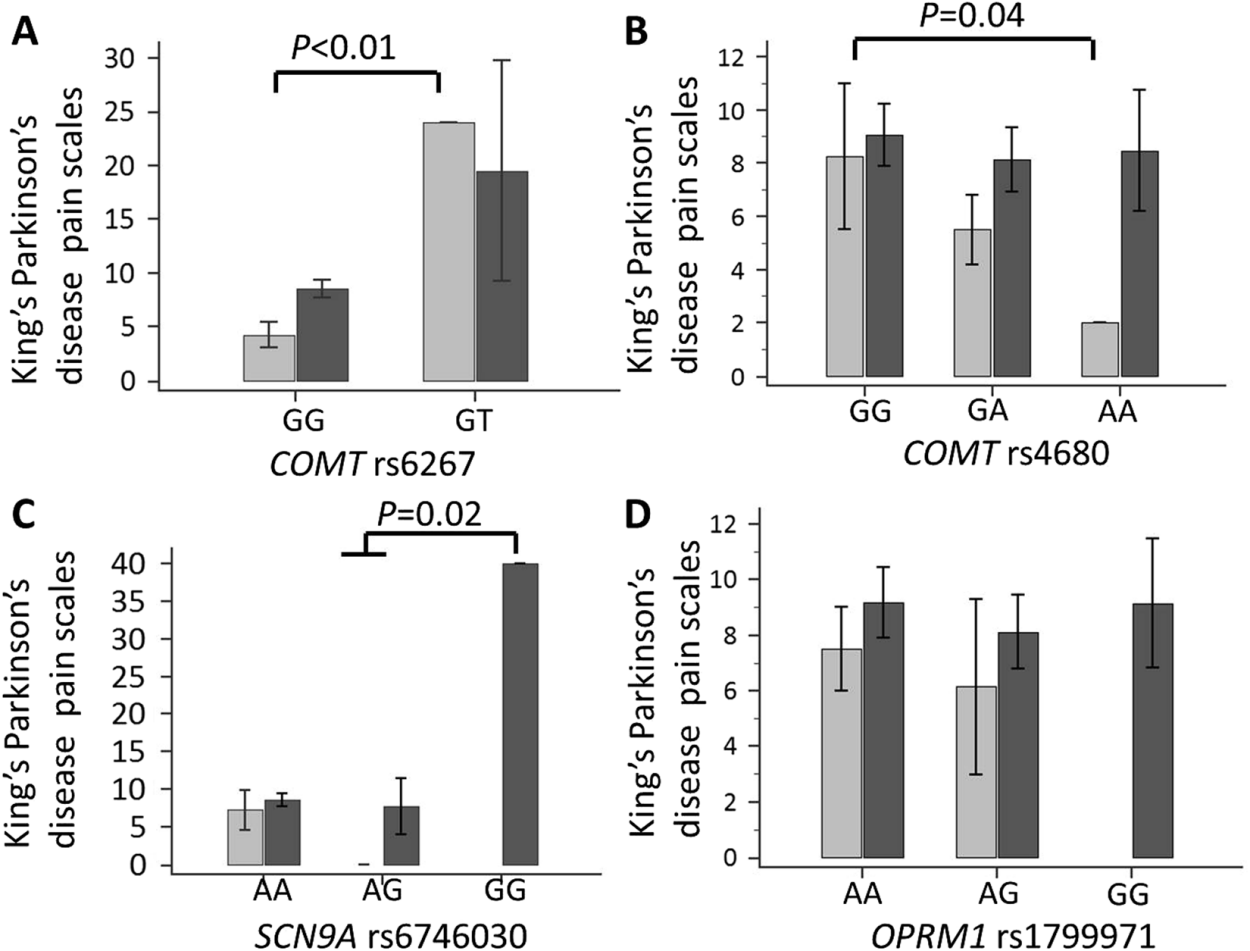
Mean KPPS scores of patients with different genotypes of *COMT* rs6267 (**A**), *COMT* rs4680 (**B**), *SCN9A* rs6746030 (**C**), and *OPRM1* rs1799971 (**D**). PD patients having different genotypes were further subgrouped into those without depression (light gray color) and those with depression (dark gray color). Data are expressed as mean + SEM. *Represents *P* < 0.05 and **represents *P* < 0.01.

Next, we examined the contribution of the SNPs of studied candidate genes and *COMT* haplotypes towards the pain severity in PD patients, we analyzed the KPSS in patients with different genotypes. We found that among patients with PD with pain, the mean pain score was significantly higher in those with the *COMT* rs6267 “GT” genotype than in those with the “GG” genotype (18.43 ± 16.9 vs. 7.89 ± 10.9, *P* = 0.01) (Fig. 3A). The pain severity was also higher in patients having the *SCN9A* rs6746030 “GG” genotype (38.9 ± 2.6) than in those having the wild-type “AA” (6.9 ± 10.2) and heterozygous “AG” (8.1 ± 11.0) genotypes (*P* = 0.02) (Fig. 3C). Pain severity was similar in patients with PD with different genotypes of the remaining *COMT* rs4680 and *OPRM1* rs1799971 (Fig. 3B,D).

Depression is an important psycho-affective contributor to the susceptibility of pain in a general population and SNPs of *COMT* gene are also reported to associate with depression^16^. We next examined the distribution of genotypes and estimated odds ratio of the studied candidate SNPs of *COMT*, *SNCA9A*, and *OPRM1* in relation to the risk of depression in our PD patients. Among 418 enrolled participants with PD, 328 patients (78.5%) were diagnosed with depression. The frequency of the *COMT* rs4680 “GA” and “AA” genotypes was modestly higher in PD patients with depression than those without depression (42.2% vs. 25.7%, *P* = 0.01, Supplementary Table 2), and the presence of the “A” allele significantly increased the risk of depression in patients with PD ([OR] = 1.89, 95% CI = 1.15–3.10, 1.89, *P* = 0.01). However, after Bonferroni correction with conservative *P* value for significance, all the studied genotype or allele frequency of *COMT* rs6267, *COMT* rs6269, *COMT* rs4633, *COMT* rs48418, *COMT* rs4680, *SNCA9A* rs6746030, and *OPRM1*rs1799971 did not significantly differ between PD patients with and without depression. In addition, while assaying the contribution of the aforementioned SNPs, *COMT* haplotypes and clinical variables in risk of depression in PD patients using a stepwise multivariate regression analysis, we found only the motor disability (UPDRS part III scores) and *COMT* rs6267 T allele contribute to the risk of depression in PD (Beta = 0.006, *P* < 0.01 for UPDRS part III scores; Beta = −0.338, *P* < 0.01 for rs6267 T allele, adjusted R^2^ = 0.067 and *P* = 0.01; Supplementary Table 3). Because depression is a strong risk factor for susceptibility of pain in PD patients (Table 2), we then stratified the analyses by participants’ depression status (Fig. 3). After stratified by depression status, the association between *COMT* rs6267 GT genotype and the intensity of pain perception still remained significant in PD patients without depression (23.32 ± 5.9 vs. 4.68 ± 10.2, *P* < 0.01; Fig. 3A). Notably, pain severity was only significantly higher in participants homozygous for *COMT* rs4680 “GG” and heterozygous for “GA” genotypes than participants homozygous for “AA” genotype in the subgroup of PD patients without depression. The effect of *COMT* rs4680 SNP in affecting pain severity was abolished in the subgroup of PD patients with depression (Fig. 3B). These findings suggest that although depression is a strong risk factor for susceptibility of pain in PD patients, *COMT* rs6267 “GT” genotype and *COMT* rs4680 “GG” and “GA” genotypes also contribute to pain susceptibility in PD patients without depression.

To further evaluate the contibutions of clinical and genetic risk factors for the severity of pain in PD patients with complaints of pain, we then applied a multivariate stepwise regression analysis incorporating onset age, disease duration, sex, depression severity, motor fuction disability and studied SNPs and *COMT* haplotypes for the KPSS socres in PD patients with pain complaints. The results showed that disease duration (beta = 0.484, *P* = 0.02), and *COMT* rs6267 “T” allele (beta = 17.154, *P* < 0.01) were significantly correlated with pain severity (R^2^ = 0.54, *P* = 0.005 for the model; Table 3). The *COMT* haplotypes consisting of allele combinations of rs6269, rs4633, rs4818 and rs4680 from 5′ to 3′ in the *COMT* gene did not significantly affect the severity of pain perception in patients with PD.

**Table 3:** Multiple linear regression analysis of factors correlated with pain severity assessed using King’s PD pain scale in patients with PD with pain (n = 193).

Independent variables	Coefficient	Std. Error	*R* partial	*t*	*P* value
(Constant)	8.092				
Age at onset (years)	−0.128	0.096	−0.110	−1.329	0.19
Disease duration (years)	0.484	0.204	0.194	2.371	0.02*
Gender	−1.353	1.843	−0.061	−0.734	0.46
BDI scores	0.177	0.119	0.123	1.489	0.14
UPDRS part III scores	0.169	0.094	0.148	1.792	0.08
rs6267 T allele	17.154	5.796	0.239	2.959	<0.01**
rs4680 A allele	0.911	3.323	0.023	0.274	0.78
rs6746030 A allele	2.411	3.473	0.058	0.694	0.49
rs1799971 G allele	0.150	1.302	0.010	0.115	0.91
Haplotype 1	0.644	2.850	0.019	0.226	0.82
Haplotype 2	−2.663	12.586	−0.017	−0.212	0.83
Haplotype 3	−3.485	3.086	−0.094	−1.129	0.26
Haplotype 4	−2.333	4.414	−0.044	−0.528	0.59
Haplotype 5	−0.063	11.945	−0.001	−0.005	0.99
Haplotype 6	−0.222	11.574	−0.002	−0.019	0.98

In this model, the presence of pain in PD patients was set as the dependent variable and the onset age of PD motor symptoms, disease duration, sex, BDI scores, UPDRS part III scores in the on state of PD, and minor allele frequency of pain-related candidate genes in this study were set as independent variables (R^2^ = 0.154 and *P* = 0.005). R: correlation coefficient based on the model of multiple linear regression; *t*: *t* value for the coefficient of each parameter in the model; *p*: for *R* or *t*. BDI, Beck Depression Inventory; UPDRS, Unified PD rating scale.

The sequence of alleles in each haplotype for central haploblock of *COMT* gene reflects the order of occurrence from 5′ to 3′ in the *COMT* gene (SNPs: rs6269, rs4633, rs4818 and rs4680, respectively). Six haplotypes out of possible 16 were detected from these four SNPs with the most frequent haplotype (Haplotype 1, 24.6%) composed of the most frequent alleles for SNPs rs4633 and rs4680 and the least frequent alleles for SNPs rs6269 and rs4818 (G_C_G_G for SNPs rs6269, rs4633, rs4818 and rs4680, respectively). The second major haplotype (Haplotype 2, 24.3%) was composed of the most frequent alleles for SNPs rs6269 and rs4818 and the least frequent alleles for SNPs rs4633 and rs4680 (A_T_C_A). The third haplotype (Haplotype 3, 16.1%) was composed of a combination of the most frequent alleles for all SNPs (A_C_C_G). The fourth major haplotype (Haplotype 4, 14.3%) composed of the least frequent alleles for all markers (G_T_G_A). The fifth major haplotype (Haplotype 5, 11.9%) composed of the described alleles of the four markers (A_C_G_G) and the sixth haploptye (Haplotype 6, 1.1%) composed of the described alleles of the four markers (G_C_C_G). These six haplotypes accounted for 92.3% of all detected haplotypes in our studied population.

## Discussion

Our study that enrolled a relatively large-sample number of PD patients with different pain status showed that depression was significantly associated with pain susceptibility in patients with PD. The *COMT* rs4680 “GA” and “AA” genotypes (Val/Met heterozygotes and Met/Met homozygotes) increased the risk of PD-associated pain. The *COMT* rs6267 “GT” genotype was associated with increased pain intensity in patients with PD with pain, even after stratified by depression status. Furthermore, the pain severity was significantly higher in participants homozygous for *COMT* rs4680 “GG” and heterozygous for “GA” genpotypes than participants homozygous for “AA” genotype in the subgroup of PD patients without depression. In addition to the exonic SNPs of *COMT*, rs6267 and rs4680, we did not find any significant associations between central *COMT* haploblock and susceptibility or intensity of pain perception in our PD population. Our findings suggested that, in addition to depression, genetic variants of *COMT* rs6267 and rs4680 can also modulate pain sensitivity in patients with PD.

Our results showed that patients with PD having homozygous “AA” (Met/Met) and heterozygous “GA” (Val/Met) genotypes of the *COMT* rs4680 are more susceptible to and have an increased risk of pain compared to those having “GG”. The *COMT* rs4680 (c.472G > A, p.V158M) is a functional exonic variant that substitutes the amino acid valine for methionine at codon 158 and determines the stability of COMT, a key metabolising enzyme for catecholamines, including dopamine and norepinephrine^17^. Its activity is mainly determined by the rs4680 (c.472G > A, p.V158M) variant of *COMT*; according its activity, COMT is categorized as a high (G/G, Val/Val), intermediate (A/G, Val/Met), and low (A/A, Met/Met) metabolizer. The relationship between the p.V158M polymorphism of *COMT* and pain sensitivity has been consistently examined in healthy individuals; the results have shown that the carriers of the “GG” (Met/Met) genotype have an increased sensitivity to painful stimuli^18, 19^ and increased brain activity over the pain-related anterior and midcingulate cortex after painful laser stimuli^20^. In addition to increasing pain susceptibility in control individuals, the met158 allele of *COMT* was found to be associated with the risk of chronic pain syndromes, such as migraine^21^, temporomandibular joint disorder^15^, and fibromyalgia^11^. These findings combined with our results in PD patients with pain suggested that *COMT* “AA” (Met/Met) variant—associated with lower COMT enzymatic activity—leads to increased pain sensitivity and that this is a common downstream nociceptive pathway in PD and other chronic pain syndromes. Laboratory evidence has shown that low COMT activity leads to increased pain sensitivity because catecholamine levels are elevated and the β-adrenergic mechanism is activated. These findings are of considerable clinical importance, suggesting that pain conditions resulting from low COMT activity or elevated catecholamine levels can be treated with pharmacological agents that block both β_2_- and β_3_-adrenergic receptors^22^. Further investigations with larger samples are required to clarify the role of *COMT* rs4680 Met/Met variant in PD pain.

In this study, we also identified depression to be a significant risk factor for pain vulnerability in patients with PD: a significant correlation was noted between BDI and KPPS scores, a correlation which persisted even after adjustments for onset age, disease duration, and genotypes of candidate genes. The mechanisms of comorbid pain and depression are well established^23^; in patients with PD, pain and depression are extremely common and frequently interrelated: chronic pain can lead to depression, and patients with depression tend to experience pain that is more intense and refractory to analgesia than do non-depressed individuals^24^. Our results corroborate previous results that patients with PD and with depression have significantly more severe pain than those without depression^24, 25^. Neuropathological studies have shown that the depression-related serotonin-producing neurons in the brainstem raphe nuclei—which have the largest and most complex efferent system in the human brain—overlap with the descending inhibitory pathway relaying pain-inhibiting messages from the brain to the spinal cord through the dorsolateral and ventrolateral funiculi^26^. Notably, these raphe nuclei and locus coeruleus containing the brainstem regions are also areas where Lewy body pathology are present in the course of PD, even from the prodromal stage according to Braak’s pathology findings^27^. This pathological evidence can partially explain the strong correlation between the severities of depression and pain in patients with PD.

Paradoxically, several studies have reported that *COMT* rs4680 p.V158M polymorphism is associated with not only pain but also a higher risk of depression^28, 29^. Depressed individuals display a higher frequency of *COMT* rs4680 Met/Met and Met/Val genotypes than do controls^29^. Our results were also in line with previous findings that A allele of *COMT* rs4680 p.V158M were modestly higher in PD patients with depression than those without depression ([OR] = 1.87, 95% CI = 1.15–3.05, *P* = 0.01), although the difference did not reach the significance level after Bonferroni correction. Furthermore, *COMT* haplotypes did not play a major role in the susceptibility of either pain or depression in our study population. We hypothesized that the relationship between depression, *COMT* polymoprphisms, especially rs4680, and pain susceptibility are complex and interrelated. To further clarify the contribution of depression and *COMT* SNPs to pain susceptibility and perception intensity in PD patients, we stratified the analyses by participants’ status of depression. After stratified by depression status, the association between *COMT* rs6267 GT genotype still remained significant in PD patients without depression. Notably, pain severity was only significantly higher in participants homozygous for *COMT* rs4680 “GG” and heterozygous for “GA” genpotypes than participants homozygous for “AA” genotype in PD patients without depression. These findings suggest that, although depression *per se* is a contributor to pain, *COMT* rs4680 and rs6267 also affect pain vulnerability in PD patients. Future studies are warranted to investigate the biological mechanisms of COMT acitivity in the pain susceptibility in PD animal models.

Our results showed that another *COMT* variant, *COMT* rs6267 (c.214 G > T, p.A72S), is significantly correlated with pain intensity, defined by KPPS scores, among patients with PD with pain. *COMT* rs6267 is a missense variant that encodes serine instead of alanine, resulting in changes in the RNA secondary structure such as decreased COMT enzymatic activity, leading to a high sensitivity to pain^30^. A study enrolling 100 patients with PD (57 with pain) also revealed that the *COMT* rs6267 T allele is associated with pain (OR = 0.216, 95% CI 0.068–0.688, *P* = 0.010)^31^. Although our results did not demonstrate an association of *COMT* rs6267 with the risk of pain in patients with PD, the mean pain score was significantly higher in the *COMT* rs6267 “GT” genotype than wild-type “GG” among patients with PD with pain. Moreover, the *COMT* rs6267 “T” allele is significantly correlated with KPPS scores after the effects of onset age, disease duration, and severities of motor symptoms and depression are considered. These findings suggest that low COMT enzymatic activity contributes to increased susceptibility and intensity of pain in patients with PD.

A strength of our study is that we included a relatively large and ethnically homogeneous sample. All our patients received regular long-term follow-up and were evaluated and diagnosed as having pain by movement disorder specialists. In addition, the confounding factors potentially influencing susceptibility to PD pain, such as age, sex, disease duration, depression symptoms, dopaminergic agent treatment, motor symptom severity, and comorbidities, were considered in the analysis. However, our study also has some limitations: although anxiety is also strongly correlated with both depression and pain, we did not include anxiety symptoms in the analysis. Second, KPPS combines scores from seven domains of PD related pain, which captures pain ranging from wearing off related pain to central, orofacial, and radicular pain. Each domain may have different genetic causes. However, because of the limited patient number in each pain domain of KPPS, we did not perform regression analysis to evaluate the association between genetic factors and the individual pain subtypes in our PD patients for the concern of statistical power. Studies with larger sample number of PD patients with different pain characters are needed to clarify the genetic risk factors for specific subtypes of PD pain in the future. Finally, our study was only focused on the PD-related pain and did not compare the results with other chronic pain syndrome, such as fibromyalgia or temporomandibular joint disorder. Future studies enrolling patients with different chronic pain syndromes are warranted to compare the effects of genetic variants of *COMT* in variable chronic pain syndrome.

In conclusion, our results reinforce previous findings that depression is a strong risk factor for pain in patients with PD. The *COMT* rs4680 and rs6267 contribute to both pain susceptibility and severity in patients with PD. Additional experimental studies are required to investigate the role of *COMT* in the nociceptive processes in patients with PD.

## Methods

### Patients

We recruited patients who fulfilled the clinical diagnostic criteria of the United Kingdom PD Society Brain Bank^32^ and were having regular follow-up at the movement disorder clinics of National Taiwan University Hospital and Chang Gung Memorial Hospital in Taiwan. Patients were excluded if their Mini–Mental State Examination scores^33^ were <24. All patients were receiving L-dopa or a combination with dopamine agonists and showed favourable clinical responses. L-dopa equivalent doses were calculated^34^. Patients maintained their regular anti-PD medications and were examined while in the “on” status. Each patient was examined using the Unified Parkinson’s Disease Rating Scale (UPDRS)^35^, modified Hoehn-and-Yahr staging^36^, Mini-Mental Status Evaluation (MMSE)^33^ and Beck depression inventory (BDI)^37^. Informed consent was obtained from all participants, and the study was approved by the institutional ethics board committees of National Taiwan University Hospital (201505003RIND) and Chang Gung Memorial Hospital (201600356A3). All methods in this study were performed in accordance with the relevant guidelines and regulations.

### Pain assessment

Two movement disorder specialists (Drs CH Lin and YR Wu) interviewed the patients by using a structured questionnaire that included demographic and clinical data, such as onset age, disease duration, and medication. In addition, patients were asked whether they experienced pain; if yes, a PD-specific pain scale, King’s PD pain scale (KPPS)^38^, and visual analogue scale were applied to describe the characteristics and severity of pain. The KPPS is a scale based on interviews conducted with patients and has seven domains with a total of 14 items^39^. The scale includes musculoskeletal pain (domain 1), chronic neuropathic pain (domain 2), motor fluctuation-related pain (domain 3), nocturnal pain (e.g., related to restless leg syndrome; domain 4), orofacial pain (domain 5), limb edema- or swelling-related pain (domain 6), and radicular pain (domain 7). Each item is scored by severity [0, (none) to 3 (extremely severe)] multiplied by frequency [0 (never) to 4 (all the time)], resulting in a subscore of 0–12, the sum of which gives the total score, with a theoretical range of 0–168. Patients who have multiple overlapping pain reports are collapsed into the major domain in the KPPS. The KPPS is currently the only validated PD-specific pain scale and is recommended by the rating scales development committee of the Movement Disorders Society for pain intensity rating^36^.

### Genetic analysis

DNA was extracted from venous blood according to standard protocols^40^. The rs6267 (c.214G > T, p.A72S), rs6269 (intronic variant G > A), rs4633 (c.435C > T, p.H62H), rs4818 (c.C657G, p.L136L) and rs4680 (c.472G > A, p.V158M) of *COMT*, rs6746030 (c.3448G > A, p.R1150W) of *SCN9A*, and rs1799971 (c.A118G, p.N40D) of *OPRM1* were genotyped by real-time polymerase chain reaction (PCR) using TaqMan^®^ Genotyping Assays on the StepOnePlus Real-Time PCR machine (Applied Biosystems). Primer sequences and PCR conditions are available on request. The NCBI SNP databases were used to assign SNP numbers.

### Statistical analysis

We tested each genetic variant for the Hardy–Weinberg equilibrium. Allele and genotype frequencies were compared between PD patients with and without pain by using the chi-squared test; alternatively, we used the Fisher exact test when the number was less than five. Numerical variables were expressed as means ± standard deviations of the means. For variables following a Gaussian distribution, data were compared using a two-tailed *t* test. We used either the Pearson product-moment correlation coefficient or Spearman rank correlation coefficient to evaluate the correlations among variables. The correlations were explored using multivariate linear regression analysis; the covariance of the model (R^2^) and the standardized correlation coefficient were presented. The potential risk factors associated with the susceptibility and severity of pain in patients with PD were examined using multivariate stepwise logistic regression analysis. Logistic stepwise regression is designed to find the most parsimonious set of predictors that are effective in predicting the dependent variable. The dependent variable in the model was the existence of symptoms of pain or KPPS score. The independent variables included in the model were age at motor symptoms onset, disease duration, sex, BDI scores, UPDRS part III scores, individual candidate genotypes and *COMT* haplotypes. The method of forward stepwise selection method with the criteria for entry of the variable is 0.05 and removal is 0.1 has been used to select the most optimal subset of independent variable. We also performed stratified analyses to evaluate potential effect modifications of depression in the susceptibility and severity of pain in patients with PD. Participants were classified according to the comorbidity of depression. A two-sided P < 0.05 was considered significant.

We performed all analyses by using Stata (StataCorp LP, College Station, USA). A *P* value was considered significant after Bonferroni correction with conservative *P* was 0.05/7 = 0.007.

## Electronic supplementary material

Supplementary Information
