# Supplementary material for: Depression and Catechol-O-methyltransferase (COMT) genetic variants are associated with pain in Parkinson’s disease
Source: Sci Rep. 2017 Jul 24;7:6306. doi: 10.1038/s41598-017-06782-z (PMC5524945; doi:10.1038/s41598-017-06782-z)
Supplement: Supplementary file 1 — Supplementary Information [file 41598_2017_6782_MOESM1_ESM.pdf]

Supplementary information:

Depression and Catechol-O-methyltransferase (*COMT*) genetic variants are associated with pain in Parkinson's disease

Chin-Hsien Lin, MD, PhD[1], K. Ray Chaudhuri, MD, DSc[2], Jun-Yu Fan, RN, PhD[3,4], Chia-I Ko, MD[1], Alexandra Rizos, MSc[5], Chia-Wen Chang MSc[6], Han-I Lin[1], Yih-Ru Wu, MD[6*]

Supplementary Figure 1: Sub-classification of PD pain according to pain subtypes of the KPPS.

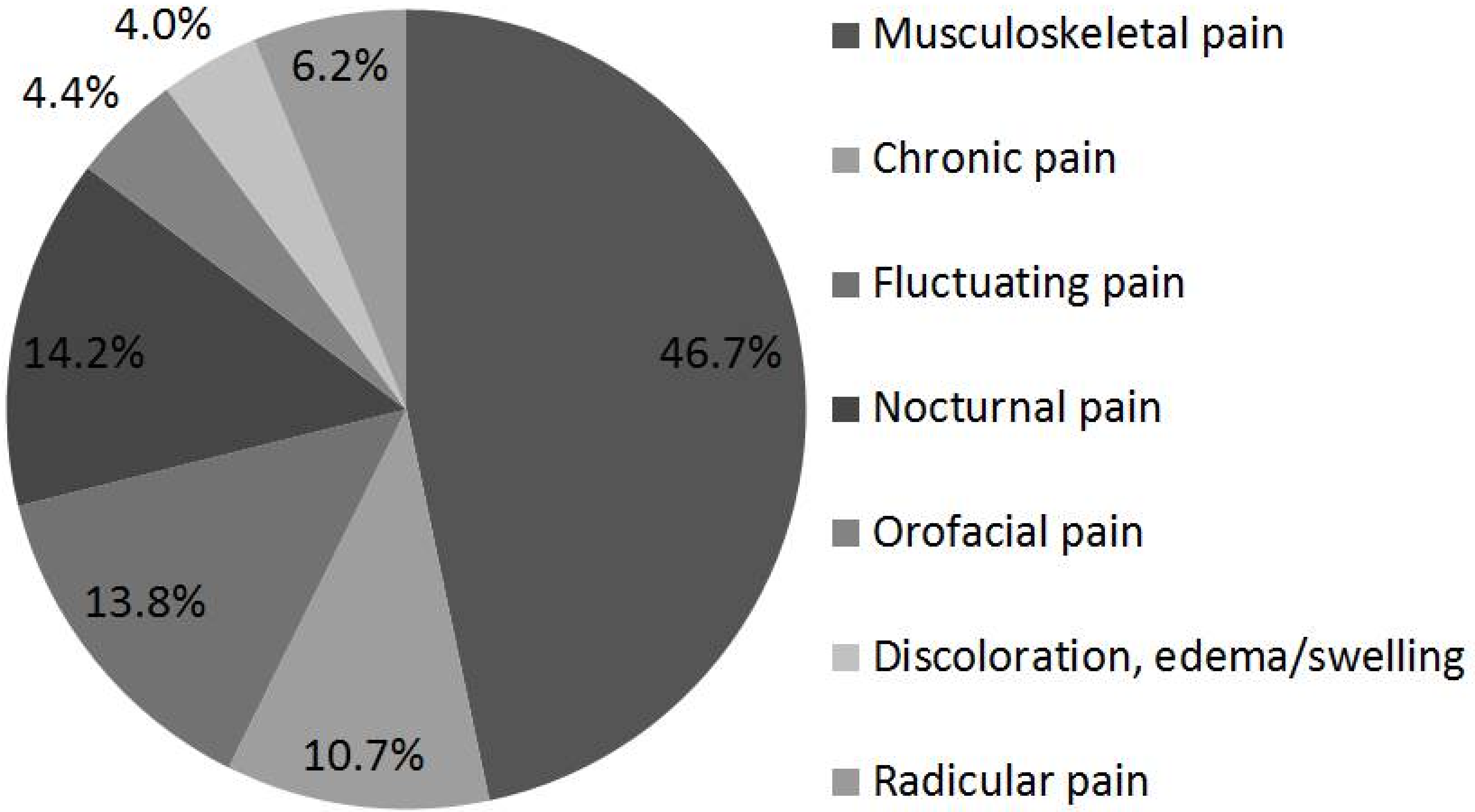

Supplementary Table 1. Clinical characteristics of patients with PD.

	PD (n=418)	Pain-free PD patients (n=225)	PD patients reported pain (n=193)	*P* Value
Current age (years)	64.5±12.6	64.7±13.3	64.5±11.1	*P*=0.87
Onset age (years)	59.6±11.3	61.4±10.8	58.5±11.2	*P*<0.01
Disease duration (years)	7.0±5.8	5.5±3.8	8.2±4.3	*P*<0.01
Gender (Male, %)	55.3	58.2	50.8	*P*=0.12
MMSE	25.9±3.7	25.8±3.4	25.9±4.2	*P* =0.78
BDI	9.7±6.9	4.6±3.8	10.6±8.9	*P*<0.01
Hoehn-Yahr stage (on)	2.0±0.9	1.9±0.9	2.2±1.0	*P*<0.01
UPDRS part I (on)	2.6±1.8	2.3±1.8	2.9±1.8	*P*<0.01
UPDRS part II (on)	11.2±6.9	8.5±5.1	13.7±7.4	*P*<0.01
UPDRS part III (on)	19.7±11.3	18.1±10.6	21.7±11.6	*P*<0.01
UPDRS part IV (on)	3.3±2.9	2.8±2.6	3.7±3.3	*P*<0.01
UPDRS total score (on)	40.2±18.4	34.2±14.7	43.8±19.6	*P*<0.01
King's PD pain scale		N.A.	11.0±8.9	
VAS		N.A.	5.8±3.2	
Daily LED (mg)		600.6±344.6	696.5±398.1	*P*<0.01
Medical co-morbidities				
Diabetes Mellitus (n, %)	49 (11.7)	25 (11.1)	24 (12.4)	*P*=0.67

Hypertension (n, %)	94 (22.5)	53 (23.6)	41 (21.2)	*P*=0.32
Osteoarthrities (n, %)	16 (3.8)	7 (3.1)	9 (4.7)	*P*=0.68
Cervical spondylosis (n,%)	4 (0.9)	3 (1.3)	3 (1.6)	*P*=0.85
Lumbar spondylosis (n, %)	6 (1.4)	6 (2.7)	5 (2.6)	*P*=0.96

PD, Parkinson disease; MMSE, Mini–Mental State Examination; UPDRS, unified PD rating scale; BDI, Beck Depression Inventory; VAS, visual analogue scale for pain; LED, levodopa equivalent dose.

Numbers are expressed as means ± standard deviations.

Supplementary Table 2. Distribution of genotypes and estimated odds ratio of genetic variants of candidate genes in relation to risk of depression in patients with PD.

	PD patients without depression N=70	PD patients with depression N=348	OR (95% CI)	*P* value[a]
COMT rs6267 c.214G>T(p.A72S)				
GG	63 (90.0%)	338 (97.1%)		
GT	7 (10.0%)	10 (2.9%)		
TT	0 (0)	0 (0)		
G vs. T allele			3.72 (0.97-14.18)	*P*=0.04
COMT rs6269 intronic variant G>A				
GG	34 (48.6%)	209 (60.0%)		
GA	36 (51.4%)	138 (39.7%)		
AA	0 (0)	1 (0.3%)		
A vs. G allele			0.73 (0.48-1.11)	*P*=0.24
COMT rs4633 c.435C>T (p.H62H)				
CC	50 (71.4%)	184 (52.9%)		
CT	17 (24.3%)	131 (37.6%)		
TT	3 (4.3%)	33 (9.5)		
T vs. C allele			2.00 (1.24-3.22)	*P*=0.03
COMT rs4818 c.C657G (p.L136L)				

CC	26 (37.1%)	168 (48.3%)		
CG	35 (50.0%)	136 (39.1%)		
GG	9 (12.9%)	44 (12.6%)		
G vs. C allele			0.78 (0.53-1.14)	*P*=0.19
COMT rs4680 c. 472G>A (p.V158M)				
GG	52 (74.3%)	201 (57.8%)		
GA	15 (21.4%)	120 (34.5%)		
AA	3 (4.3%)	27 (7.7%)		
A vs. G allele			1.89 (1.15-3.10)	*P*=0.01
SCN9A rs6746030 c.3448G>A (p.R1150W)				
GG	66 (94.3%)	330 (94.8%)		
GA	4 (5.7%)	17 (4.9%)		
AA	0 (0)	1 (0.3%)		
A vs. G allele			0.95 (0.32-2.85)	*P*=0.93
OPRM1 rs1799971 c.A118G (p.N40D)				
AA	27 (38.5%)	172 (49.4%)		
AG	32 (45.8%)	125 (35.9%)		
GG	11 (15.7%)	51 (14.7%)		
G vs. A allele			0.77 (0.53-1.12)	*P*=0.17

PD, Parkinson disease; OR, odds ratio; CI, confidence interval.

[a]Chi-Square test or Fisher exact test (when frequency < 5) were applied.

**P* value remained significant after Bonferroni correction with conservative *P* = 0.05/7 = 0.007.

Supplementary Table 3. Multiple stepwise regression analysis of factors correlated with susceptibility of depression in patients with PD (n = 418).

Independent variables	Coefficient	Std. Error	*R* partial	*t*	*P* value
(Constant)	0.891				
Age at onset (years)	-0.002	0.002	-0.046	-0.652	0.52
Disease duration (years)	-0.002	0.004	-0.034	-0.480	0.63
Gender	0.016	0.041	0.029	0.398	0.69
UPDRS part III scores	0.006	0.002	0.214	3.042	0.003**
rs6267 T allele	-0.338	0.098	-0.242	-3.448	0.007**
rs4680 A allele	0.005	0.072	0.005	0.073	0.94
rs6746030 A allele	-0.016	0.081	-0.014	-0.199	0.84
rs1799971 G allele	-0.043	0.027	-0.113	-1.567	0.12
Haplotype 1	0.004	0.064	0.004	0.058	0.95
Haplotype 2	-0.109	0.198	-0.039	-0.553	0.58
Haplotype 3	0.002	0.070	0.002	0.025	0.97
Haplotype 4	-0.045	0.093	-0.035	-0.482	0.63
Haplotype 5	0.166	0.179	0.067	0.929	0.35
Haplotype 6	0.061	0.294	0.015	0.209	0.83

In this model, the presence of depression in patients with PD was set as the dependent variable and the onset age of PD motor symptoms, disease duration, sex, UPDRS part III scores in the on state of PD, and minor allele frequency of pain-related candidate genes in this study were set as

the independent variables (R^2 = 0.067 and P = 0.01). R: correlation coefficient based on the model of logistic regression; *t*: *t* value for the coefficient of each parameter in the model; *p*: for *R* or *t*. The sequence of alleles in each haplotype for central haploblock of *COMT* gene reflects the order of occurrence from 5' to 3' in the *COMT* gene (SNPs: rs6269, rs4633, rs4818 and rs4680, respectively). Six haplotypes out of possible 16 were detected from these four SNPs with the most frequent haplotype (Haplotype 1, 24.6%) composed of the most frequent alleles for SNPs rs4633 and rs4680 and the least frequent alleles for SNPs rs6269 and rs4818 (G_C_G_G for SNPs rs6269, rs4633, rs4818 and rs4680, respectively). The second major haplotype (Haplotype 2, 24.3%) was composed of the most frequent alleles for SNPs rs6269 and rs4818 and the least frequent alleles for SNPs rs4633 and rs4680 (A_T_C_A). The third haplotype (Haplotype 3, 16.1%) was composed of a combination of the most frequent alleles for all SNPs (A_C_C_G). The fourth major haplotype (Haplotype 4, 14.3%) composed of the least frequent alleles for all markers (G_T_G_A). The fifth major haplotype (Haplotype 5, 11.9%) composed of the described alleles of the four markers (A_C_G_G) and the sixth haploptye (Haplotype 6, 1.1%) composed of the described alleles of the four markers (G_C_C_G). These six haplotypes accounted for 92.3% of all detected haplotypes in our studied population.
